# miR-150 Deficiency Protects against FAS-Induced Acute Liver Injury in Mice through Regulation of AKT

**DOI:** 10.1371/journal.pone.0132734

**Published:** 2015-07-21

**Authors:** Weina Chen, Chang Han, Jinqiang Zhang, Kyoungsub Song, Ying Wang, Tong Wu

**Affiliations:** 1 Department of Pathology and Laboratory Medicine, Tulane University School of Medicine,1430 Tulane Avenue SL-79, New Orleans, Louisiana, United States of America; 2 Department of Gastroenterology and Internal Medicine, Tongji Hospital, Tongji Medical College, Huazhong University of Science and Technology, Wuhan, China; CIMA. University of Navarra, SPAIN

## Abstract

Although miR-150 is implicated in the regulation of immune cell differentiation and activation, it remains unknown whether miR-150 is involved in liver biology and disease. This study was performed to explore the potential role of miR-150 in LPS/D-GalN and Fas-induced liver injuries by using wild type and miR-150 knockout (KO) mice. Whereas knockout of miR-150 did not significantly alter LPS/D-GalN-induced animal death and liver injury, it protected against Fas-induced liver injury and mortality. The Jo2-induced increase in serum transaminases, apoptotic hepatocytes, PARP cleavage, as well as caspase-3/7, caspase-8, and caspase-9 activities were significantly attenuated in miR-150 KO mice. The liver tissues from Jo2-treated miR-150 KO mice expressed higher levels of Akt1, Akt2, total Akt, as well as p-Akt(Ser473) compared to the wild type livers. Pretreatment with the Akt inhibitor V reversed Jo2-induced liver injury in miR-150 KO mice. The primary hepatocytes isolated from miR-150 KO mice also showed protection against Fas-induced apoptosis *in vitro* (characterized by less prominent PARP cleavage, less nuclear fragmentation and less caspase activation) in comparison to hepatocytes from wild type mice. Luciferase reporter assays in hepatocytes transfected with the *Akt1* or *Akt2* 3’-UTR reporter constructs (with or without mutation of miR-150 binding site) established *Akt1* and *Akt2* as direct targets of *miR-150*. Tail vein injection of lentiviral particles containing pre-*miR-150* enhanced Jo2-induced liver injury in miR-150 KO mice. These findings demonstrate that miR-150 deficiency prevents Fas-induced hepatocyte apoptosis and liver injury through regulation of the Akt pathway.

## Introduction

MicroRNAs (miRNAs) are a class of small noncoding RNA molecules of about 19–25 nucleotides in length that regulate target gene expression at post-transcriptional level. miR-150 has recently been identified as a key regulator of immune cell differentiation and activation, as it is preferentially expressed in mature B and T cells as well as NK cells and other cell types of the hematopoietic system including megakaryocytes[1–7]. It was reported that miR-150 was associated with chronic lymphocytic leukemia disease[8–10]. Although miR-150 was initially appreciated as a key miRNA in hematopoietic and immune cells, emerging evidence suggests that it also mediates cell functions outside of the hematopoietic system. For example, miR-150 has recently been reported to inhibit the growth and survival of pancreatic cancer cells[11, 12]. miR-150 is implicated in the maturation of endothelial progenitor cells[13] in the regulation of endothelial cell migration[14]. miR-150 down-regulation also contributes to the constitutive type I collagen overexpression in scleroderma dermal fibroblasts via the induction of integrinβ3[15].

In the liver, miR-150 has been reported to regulate the production of type I and V collagen by hepatic stellate cells[16]. MiR-150 is downregulated in hepatic stellate cells during liver fibrosis and its overexpression causes decreased hepatic stellate cell activation[17]. Interestingly, the expression of miR-150 is found to be higher in CD133- compared to CD133+ HCC stem-like cells[18]. The level of circulating miR-150 is increased in patients with hepatocellular carcinoma[19] and intrahepatic cholangiocarcinoma[20].

Consistent with the role of miR-150 in regulation of immune response, miR-150 has been implicated in host response to the endotoxin lipopolysaccharide (LPS). For example, injection of LPS into healthy humans causes reduction of miR-150 in leukocytes[21]. Patients with sepsis show reduced plasma levels of miR-150[22]; low miR-150 serum level is associated with an unfavorable prognosis of patients with hepatic or renal dysfunction[23]. Accordingly, circulating miR-150 is emerging as a potential prognostic marker in assessing sepsis and associated organ damage[23].

Given the documented importance of LPS in the pathogenesis of liver diseases and liver injuries[24] and the emerging role of miR-150 in LPS-initiated response, we utilized miR-150 ΚΟ mice to determine whether miR-150 might influence LPS/D-GalN- and Fas-induced liver injuries. Our data showed that the miR-150 KO and wild type (WT) mice did not differ significantly in liver injury after LPS/D-GalN injection. Surprisingly, we noticed that miR-150 deficiency protected against Fas-induced liver injury. Our further experiments indicated that the primary hepatocytes isolated from miR-150 KO mice also protected against Fas-induced apoptosis, *in vitro*. The current paper details our experimental findings in LPS- and Fas-induced acute liver injuries in the miR-150 KO mice.

## Materials and Methods

### Ethical Statement

All animal procedures were carried out in strict accordance with the National Institutes of Health Guidelines for the Care and Use of Laboratory Animals. The handling of the mice and all experimental procedures were specifically approved for this study by the Institutional Animal Care and Use Committee of Tulane University. For survival experiments, humane endpoints were applied to this study. For liver sample collection, all the mice were sacrificed by CO_2_ asphyxiation. All efforts were made to minimize the distress and suffering of the animals.

### Bioinformatics analysis

The mature miR-150 sequence in mouse, human, rat, dog and gorilla was downloaded from http://www.mirbase.org. According to the paper previously described[25], the conservation alignment for the miR-150 was performed using USSC Genome Bioinformatics (http://genome.ucsc.edu). To predict the potential targets of miR-150, the following bioinformatics prediction systems were used: http://www.microRNA.org, http://www.targetscan.org, http://www.mirbase.org, and RNA22 microRNA prediction algorithm[26].

### Animal experiments

C57BL/6 wild type (WT) mice and miR-150 knockout (KO) mice were obtained from the Jackson Laboratory (Bar Harbor, ME), and the colonies were maintained at the Tulane University Health Sciences Center Animal Facility. The mice were kept at 22°C under a 12-hour light/dark cycle and received food and water freely. Eight-week-old male C57BL/6 WT and miR-150 KO mice were used in this study.

For survival experiments, the mice were injected intraperitoneally with 0.35 μg/g of body weight (BW) Jo2 (anti-Fas antibody) (BD Bioscience, Franklin Lakes, NJ) to determine the survival rate. Jo2 was dissolved in sterile 1× Dulbecco’s Phosphate Buffered Saline (Sigma-Aldrich St. Louis, MO). The mice were observed every one hour for up to 24 hours after Jo2 injection. The moribund states were used as humane endpoints. The following criteria have been used to identify moribund states: a) Inability to roll over from side to chest; b) Dyspnea, Labored breathing; c) Hypothermia. Once the mice showed the signs of moribund states, the mice were euthanized by CO_2_ asphyxiation and the death time was recorded. After Jo2 injection, twelve C57BL/6 WT mice and nine miR-150 KO mice showed moribund states and euthanized by CO_2_ asphyxiation; three miR-150 KO mice survived.

To assess the extent of Jo2-induced liver injury, the mice were intraperitoneally administered Jo2 0.5 μg/g of body weight and the animals were humanely sacrificed by CO_2_ asphyxiation at indicated time points. The liver tissues were rapidly excised and the specimens were immediately cut into small fragments and subjected to standard formalin fixation for histological evaluation as we previously described[27]. The remaining liver samples were immediately frozen in liquid nitrogen and stored at -80°C for the future preparation of tissue homogenates. Blood was collected from mouse orbital sinus according to the standard procedure[28]. The blood samples were centrifuged at 3000× rpm for 15 minutes and the sera were collected and stored at -80°C. Serum alanine aminotransferase (ALT) and aspartate aminotransferase (AST) levels were measured with an automatic analyzer at the Department of Clinical Chemistry, Tulane University Hospital.

For lipopolysaccharide (LPS) induced liver injury, mice were administered intraperitoneally with 30ng/g body weight LPS (*Escherichia coli* O55:B6; Sigma-Aldrich, St. Louis, MO) in combination with 800 μg/g body weight of D-galactosamine (D-GalN) (Sigma-Aldrich, St. Louis, MO) (LPS and D-GalN were dissolved in a sterile, nonpyrogenic 0.9% Sodium Chloride Injection). To determine the survival rate, the mice were observed every one hour for up to 24 hours after LPS/D-GalN injection. The moribund states were used as humane endpoints. Once the mice showed the signs of moribund states, the mice were euthanized by CO_2_ asphyxiation and the death time was recorded. To assess the extent of LPS/D-GalN-induced liver injury, the animals intraperitoneally injected with 30ng/g body weight LPS in combination with 800 μg/g body weight D-GalN were sacrificed by CO_2_ asphyxiation at 4 hours after LPS/D-GalN administration. The blood samples and liver tissues were collected.

We used lentiviral vector to restore miR-150 expression in miR-150 KO mice. Purified lentiviral particles containing pre-*miR-150* or scrambled control miRNA were obtained from GeneCopoeia (Rockville, MD, USA). Eight-week-old miR-150 KO mice were administered lentiviral particles containing pre-*miR-150* (LV-*miR-150*, 1.01×10^9^ copies/mouse), or lentiviral particles containing scrambled miRNA (LV- scrambled-miRNA, 4.8×10^8^ copies/mouse) by tail vein injection in a volume of 200 μL of sterile saline. One week after virus injection, the mice were intraperitoneally administered Jo2 0.5 μg/g of body weight. 4 hours after Jo2 injection, the mice were sacrificed by CO_2_ asphyxiation and blood/liver tissue samples were collected for further analyses.

### Hematoxylin and Eosin Staining

Liver tissues were fixed in 10% buffered formalin and embedded in paraffin. Sections of 5 μm thickness were affixed to slides, deparaffinized, and stained with hematoxylin and eosin. Light microscopy was performed to determine the morphological changes.

### Cleaved caspase-3 staining

Liver tissue sections were stained with cleaved caspase-3 antibody (Biocare Medical, Pike Lane Concord, CA) which specifically recognizes the large fragment (17/19 kDa) of activated caspase-3. Specifically, formalin fixed slides (5 μm thick) were deparaffinized in xylene and hydrated in ethanol; the sections were processed for heat-induced epitope retrieval under pressure by using Biocare's Decloaking Chamber (Biocare Medical, Pike Lane Concord, CA). Following blocking with Biocare's Peroxidazed 1 for 5 minutes, the slides were incubated for 1 hour at room temperature with cleaved caspase-3 rabbit polyclonal antibody. The slides were then washed with TBS and incubated with horseradish peroxidase (HRP) conjugated goat anti-rabbit IgGs for 1 hour at room temperature. After washing with TBS, DAB (3, 3'-diaminobenzidine) HRP substrate was added, producing a brown chromogenic reaction product.

### TUNEL staining

DeadEnd Colorimetric TUNEL System (Promega Corporation, Madison, WI) was used to examine apoptotic cell death of the liver tissue. 5 μm thick, formalin fixed slides were washed twice in xylene (5 minutes each time), hydrated in 100% ethanol for 2 minutes, and washed in decreasing concentrations of ethanol (100%, 95% and 80%) (2 minutes each time). The slides were then immersed in H_2_O for 2 minutes. Terminal deoxynucleotidyl transferase-mediated dUTP nick-end labeling (TUNEL) was performed according to the manufacturer’s instructions.

### Caspase activities assays

Cytosolic extracts from liver tissue were prepared by Dounce homogenization in hypotonic extraction buffer as previous described[29]. The samples were analyzed for caspase-3/7, caspase-8 and caspase-9 activities using the Caspase-Glo Assay Kit (Promega Corporation, Madison, WI).

### Quantitative real-time polymerase chain reaction (qRT-PCR)

Total RNAs from various tissues (heart, liver, spleen, lung and kidney) were isolated from eight-week-old male WT and miR-150 KO mice by Trizol (Invitrogen, Grand Island, NY). For first-strand complementary DNA synthesis, 1 μg total RNA was reverse-transcribed using Qiagen miScript reverse transcription kit (Qiagen, Valencia, CA). Qiagen miScript SYBR green PCR kit and miR-150 specific miScript Primer Assay were used to amplify the mature form of miR-150 on Bio-Rad C1000 Thermal Cycler (Hercules, CA). The miScript II RT Kit, together with the miScript PCR System, ensures sensitive, specific miRNA detection and quantification. MiR-150 was quantified and normalized with the internal control U6 snRNA.

### Hepatocyte isolation and culture

Hepatocytes were isolated from eight-week-old male WT and miR-150 KO mice by an adaptation of the calcium two-step collagenase perfusion technique as we described previously[30]. Cells were plated onto collagen-coated 6-well plates at a density of 1×10^6^ cells per well or 10cm dishes at a density of 3×10^6^ cells (collagen I coated plates and dishes were purchased form BD Biosciences, San Jose, CA). Hepatocytes were maintained in Williams’ Medium E medium (Invitrogen, Grand Island, NY) supplemented with Hepatocyte Maintenance Supplement Pack (Invitrogen, Grand Island, NY), 10% fetal calf serum (Sigma-Aldrich St. Louis, MO), 2mM L-Glutamine (Invitrogen, Grand Island, NY) and Antibiotic- Antimycotic (Invitrogen, Grand Island, NY). After 2 hours of incubation to allow attachment, the hepatocyte cultures were washed with PBS and incubated with media containing 0.5 μg/mL Jo2 plus 10 μg/mL cycloheximide (CHX) (Sigma-Aldrich St. Louis, MO). Four hours after treatment, the cell lysates were obtained for caspase activity assays and Western blotting.

### Luciferase reporter assay

The 3’ untranslated region (UTR) of mouse *Akt1* and *Akt2* were cloned downstream of the firefly luciferase reporter gene in pEZX-MT01 vector (GeneCopoeia, Rockville, MD, USA). These vectors also express the Renilla luciferase serving as internal controls for the dual-luciferase assay. Mutated *Akt1* and *Akt2* plasmid were constructed using the Quick Change II XL Site-Directed Mutagenesis Kit (Agilent Technologies, Santa Clara, CA). The mutagenic primers of *Akt1* were: 5’-GCGGGTACCCTGGCTGCGCCTGCCTCAC-3’ (forward) and 5’-GTGAGGCAGGCGCAGCCAGGGTACCCGC-3’ (reverse). The mutagenic primers of *Akt2* were: 5’-GGGACAGGCTGTTGGCTGGATGGAGAGTGAGG-3’ (forward) and 5’-CCTCACTCTCCATCCAGCCAACAGCCTGTCCC-3’(reverse). Hepatocytes were isolated from WT mice and plated onto collagen-coated 6-well plates at a density of 1×10^6^ cells per well. After 2 hours of incubation to allow attachment, hepatocytes were transfected with miR-150 mimic or control miRNA mimic using Targefect F2 plus Virofect enhancer (Targetingsystems, El Cajon, CA). Specifically, 4 μL of Virofect enhancer (Targetingsystems, El Cajon, CA) was first added to 800 μL of culture medium in each well; following 2 h incubation, 200μL of medium containing 2μL of Targefect F2 (Targetingsystems, El Cajon, CA) and 50nM miR-150 mimic- (Qiagen, Valencia, CA) or control scrambled miRNA (Qiagen, Valencia, CA) was added. Twelve hours after miRNA transfection, the hepatocytes were transfected with 1 μg of the 3’UTRs of *Akt1* or *Akt2* plasmids or their corresponding mutants using Targefect-Hepatocyte reagent (Targetingsystems, El Cajon, CA). The cells were harvested 24 hours after plasmid transfection and analyzed by using Dual-Luciferase reporter assay system (Promega, Madison, WI). Luciferase activity was measured by centro xs3 lb 960 microplate fluorescence reader (Mandel, Ontario, Canada).

### Hoechst staining

Hoechst 33342 (Thermo, Grand Island, NY) was used to assess compacted nuclear chromatin and apoptosis. Hepatocytes were isolated from eight-week-old male WT mice and miR-150 KO mice. The isolated hepatocytes were plated onto collagen-coated 6-well plates (BD Biosciences, San Jose, CA) at a density of 1×10^6^ cells per well. After 2 hours of incubation to allow attachment, the hepatocytes were exposed to the medium with or without Jo2 0.5 μg/mL plus CHX 10 μg/mL for 4 hours. Following washing with PBS, the cells were incubated with Hoechst 33342 (1 μg/mL in 1× DPBS) for 30 minutes at room temperature in the dark. The nuclear morphology was observed at 200× under fluorescence microscope using a wavelength at 460 to 490 nm.

### Western blot analysis

To isolate proteins from liver tissues, the tissue samples were lysed with NP-40 lysis buffer [1% NP-40, 0.5% sodium deoxycholate, 0.1% sodium dodecyl sulfate phosphate-buffered saline, PH7.4; one tablet of protease inhibitor cocktail (Roche, Indianapolis, IN) and one tablet of phosphatase inhibitor cocktail (Roche, Indianapolis, IN) were added per 10 mL lysis buffer]. The produced liver homogenates were centrifuged at 12,000× g for 20 minutes at 4°C and the supernatants were collected for Western blotting. To isolate proteins from cultured hepatocytes, the cells were washed twice with ice-cold 1×PBS and lysed in a lysis buffer containing 50 mM HEPES, 1 mM EDTA (PH 8.0) [One tablet of protease inhibitor cocktail (Roche, Indianapolis, IN) and one tablet of phosphatase inhibitor cocktail (Roche, Indianapolis, IN) were added per 10 mL lysis buffer]. After sonication on ice, the cell lysates were centrifuged at 12,000× g for 20 minutes at 4°C and the supernatants were collected for Western blotting. The protein concentration was measured with Bio-Rad Protein Assay Kit (Bio-Rad, Hercules, CA). After boiling at 95°C for 5 min in the protein loading buffer with 2- mercaptoethanol, the samples were separated on a 10% sodium dodecyl sulfate polyacrylamide gel electrophoresis (SDS-PAGE) gel and then transferred onto the nitrocellulose membrane (Bio Rad, Hercules, CA). The membranes were blocked in 5% nonfat milk (in 1×PBS) for 1 hour. Blots were incubated with primary antibodies in 5% nonfat milk (in 1×PBS) at 4°C overnight. After 3 washes with PBS-T (0.1% Tween 20 in 1×PBS), the membranes were incubated with secondary antibody (horseradish peroxidase-conjugated IgG) in 5% nonfat milk (in 1×PBS) at room temperature for 1 hour. The bands were analyzed by LI-COR Odyssey (Lincoln, NE). The primary antibodies against total Akt1, Akt2, total Akt, p-Akt(Ser473), 4E-BP1, p-4E-BP1(Thr37/46), PARP, caspase-3, cleaved caspase-8 and caspase-9 were obtained from Cell Signaling Technology (Beverly, MA); the antibody against GAPDH was obtained from Ambion (Austin, TX); goat anti-rabbit or goat anti-mouse second antibodies were purchased from LI-COR Biosciences (Lincoln, NE).

### Statistical analysis

Data are presented as mean ± SD; differences between two groups were determined by two tailed Student’s t test using Microsoft Excel spreadsheet software (Microsoft Corp., Redmond, WA). Kaplan-Meier survival analysis (log-rank) was utilized for mortality analysis using SPSS for Windows software, version 13.0 (SPSS Inc., Chicago, IL). A value of *p*<0.05 was considered to be statistically significant.

## Results

### Deletion of miR-150 does not prevent LPS-induced liver injury

**Fig 1:**
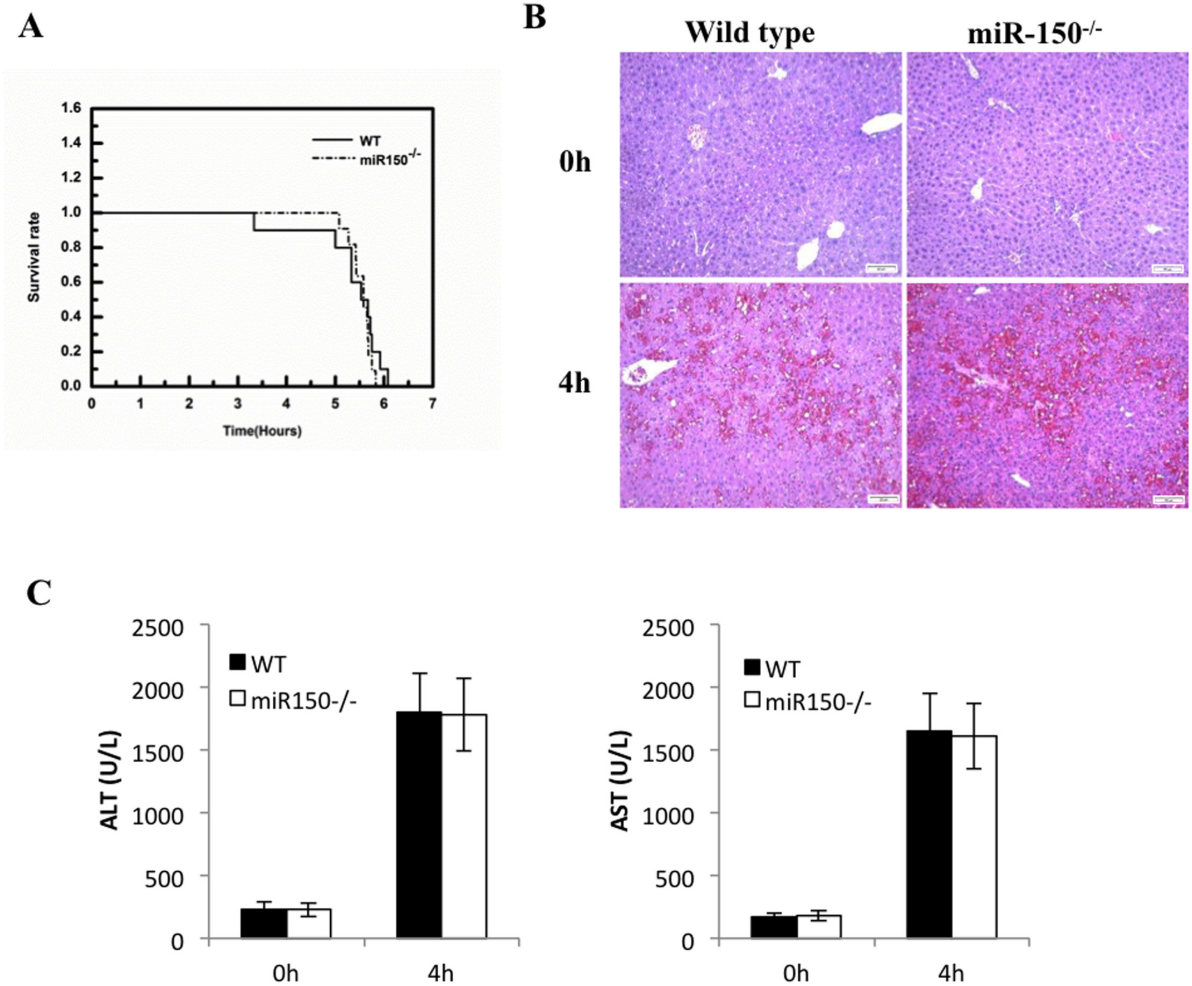
miR-150 KO fails to protect mice against LPS/D-GalN induced liver injury. (A) WT and miR-150 KO mice were intraperitoneally injected with LPS/GalN (30ng/g body weight LPS in combination with 800 μg/g body weight of D-GalN) (n = 12 per group). Survival rates are shown after LPS/GalN treatment. There was no difference between these two groups. (B) H&E staining (100×, scale bar 20μm). WT and miR-150 KO mice were intraperitoneally injected with LPS/GalN (30ng/g body weight LPS in combination with 800 μg/g body weight of D-GalN) (n = 6 per group). Mice were sacrificed 0 and 4 hours after LPS/D-GalN injection. (C) Serum levels of ALT and AST at 4 hours after LPS/D-GalN treatment. Data expressed as mean ±SD.

To determine the potential role of miR-150 in LPS-induced liver injury, we utilized a mouse model of acute liver injury induced by LPS plus D-Galactosamine (D-GalN). Specifically, 8 weeks old male C57BL/6 WT and miR-150 KO mice were injected intraperitoneally with LPS/D-GalN (30 ng/g body weight of LPS in combination with 800 μg/g body weight of D-GalN). Both the WT and miR-150 KO groups of mice died at a similar time frame (within approximately 6 hours) after LPS/D-GalN challenge. There was no significant difference in the mortality between these two groups (Fig 1A). Histopathological examination of the liver tissues from the WT mice and miR-150 KO mice showed similar degree of liver damage (Fig 1B). There was no significant difference in the levels of serum ALT and AST between the WT and miR-150 KO groups (Fig 1C). Thus, miR-150 deficiency is unable to prevent LPS/D-GalN-induced liver injury.

### Deletion of miR-150 prevents Fas-induced liver injury

**Fig 2:**
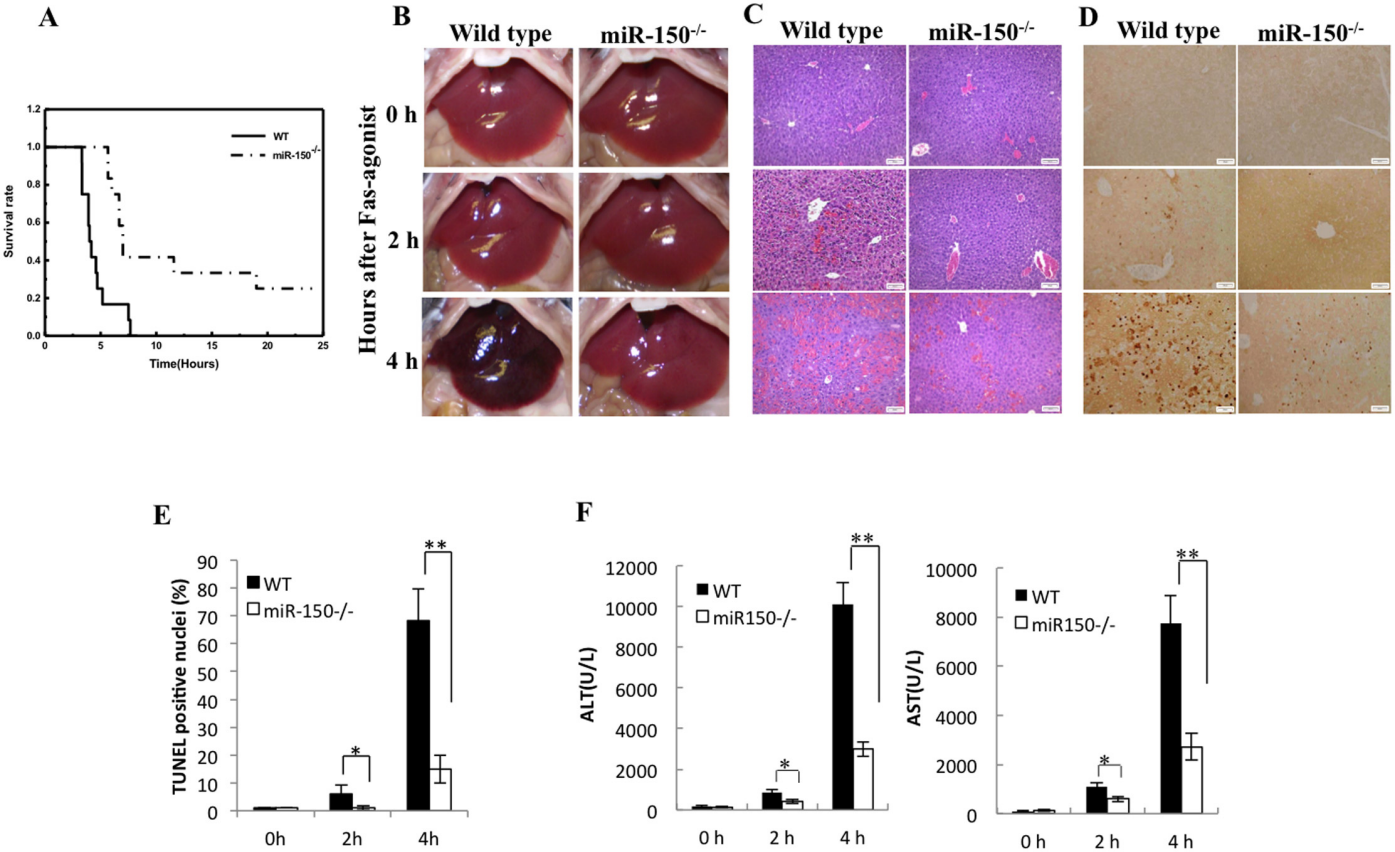
miR-150 deficiency prevents Fas-induced liver injury. (A) WT and miR-150 KO mice were intraperitoneally injected with 0.35 μg/g of body weight Jo2 (n = 12 per group). Survival rates are shown after Jo2 treatment. The mortality of WT mice was significantly higher compared to the miR-150 KO mice (*P*<0.01, log-rank test). (B) WT and miR-150 KO mice were intraperitoneally injected with 0.5 μg/g of body weight Jo2 (n = 6 or 8 per group, respectively). Gross photographs of livers were taken 0, 2 and 4 hours after Jo2 injection. There is no difference between WT and miR-150 KO mice of control group and after 2h Jo2 treatment. After Jo2 treatment for 4h, the livers of WT mice turn dark red because of massive hepatic hemorrhaging, the livers of miR-150 KO mice only showed slightly red. (C) The mice were sacrificed 0, 2 and 4 hours after injection of 0.5 μg/g of body weight Jo2. Formalin-fixed and paraffin-embedded liver tissue sections (5 μm thick) were stained with Hematoxylin and eosin (H&E, 100×, scale bar 20μm). (D) TUNEL analysis of liver tissues from WT and miR-150 KO mice 2 and 4 hours after Jo2 injection. Representative photographs (100×, scale bar 20μm) are shown. (E) Quantification of TUNEL-positive hepatocytes (Data are expressed as mean ± SD, *p<0.05, **p<0.01). (F) Serum levels of ALT and AST (0, 2 and 4 hours after injection of 0.5 μg/g of body weight Jo2). The miR-150 KO mice showed significantly lower levels of serum ALT and AST than the WT mice after Jo2 treatment. The data are expressed as mean ± SD, *p<0.05, *p<0.01.

We next utilized a separate model of acute liver injury induced by Fas activation. In this protocol, the miR-150 KO mice and age/sex matched WT mice were injected intraperitoneally with a single dose of Jo2 (0.35 μg/g of body weight). After Jo2 challenge, the mice were monitored continuously up to 24 hours. We observed that the mortality of WT mice was higher compared to the miR-150 KO mice (*p*<0.01, Fig 2A). While all of the wild type mice became lethargic and died within 10 hours, approximately 25% miR-150 KO mice were alive (3/12 miR-150 KO mice survived). To assess the degree of liver injury, separate groups of mice were treated with Jo2 and the animals were sacrificed at indicated time points (2 and 4 hours). As shown in Fig 2B, although at 2 h the livers of wild type and miR-150 KO mice had similar gross appearance, at 4 h the livers of the WT mice turned dark red due to massive hepatic hemorrhaging whereas the livers of the miR-150 KO mice only appeared slightly red. Histological examination showed less liver tissue injury in miR-150 KO mice compared to the wild type mice. Under H&E staining, the WT livers showed prominent hepatocyte apoptosis and dropout with parenchymal collapse, massive hemorrhage and congestion, whereas the miR-150 KO livers showed much less degree of hepatocyte apoptosis with only mild congestion and no significant parenchymal collapse (Fig 2C). TUNEL staining of the liver tissue sections showed less hepatocyte apoptosis in the miR-150 knockout mice compared to the wild type mice (Fig 2D and 2E). The levels of serum ALT and AST in the miR-150 KO mice were significantly lower compared to the wild type mice (Fig 2F). These findings indicate that miR-150 knockout ameliorates Fas-induced hepatocyte apoptosis and liver injury.

### Deletion of miR-150 prevents Fas-induced activation of caspase-3/7, 8 and 9

**Fig 3:**
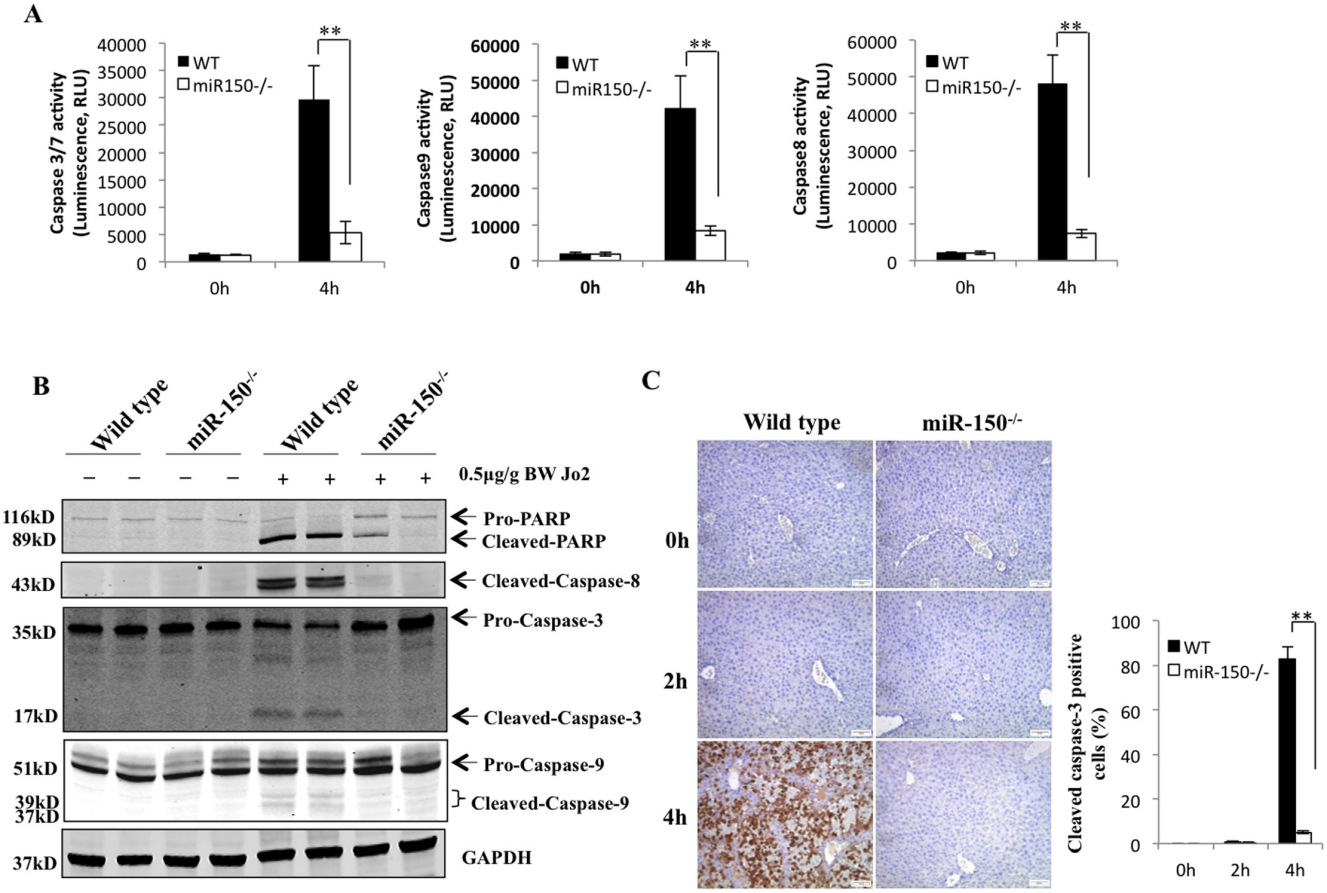
miR-150 deficiency protects against Fas-induced caspase and PARP cleavage. WT and miR-150 KO mice were intraperitoneally injected with 0.5μg/g of body weight Jo2 (n = 6 or 8 per group, respectively). The animals were sacrificed 0, 2 and 4 hours after Jo2 injection. (A) Caspase activity in liver tissue 4 hours after Jo2 injection. The levels of caspase-3/7, caspase-8, and caspase-9 activities in miR-150 KO livers were significantly lower compared to the WT livers (the data are expressed as mean ± SD) **p<0.01. (B) Western blotting analysis to detect PARP and caspases cleavage. (C) Formalin-fixed and paraffin-embedded sections (5 μm thick) were stained with antibody against cleaved caspase-3 (100×, scale bar 20μm). miR-150 KO livers showed fewer numbers of caspase- 3-positive hepatocytes compared to the WT livers (4 hours after Jo2 treatment). Quantification of cleaved caspase-3 positive hepatocytes is shown at the right panel (data are expressed as mean ± SD, **p<0.01).

Fas is known to activate caspase-8 which leads to further activation of caspase-3/7 and caspase-9. Given the involvement of these caspases in Fas-induced apoptosis, we next measured their activities in the livers from the WT and miR-150 KO mice after Jo2 treatment. As shown in Fig 3A, the activities of caspase-3/7, caspase-8 and caspase-9 in the miR-150 KO livers were lower compared to the WT livers. These findings were further corroborated by the Western blot analyses showing decreased cleavage of caspase-3, caspase-8, caspase-9 and PARP in miR-150 KO livers (Fig 3B). Decreased caspase-3/7 activation in miR-150 KO livers was also confirmed by the immunohistochemical staining (Fig 3C). These results suggest that miR-150 deficiency prevents Fas-induced caspase activation in the liver.

### Increased expression of Akt1, Akt2, total Akt and p-Akt(Ser473) in miR-150 KO livers

**Fig 4:**
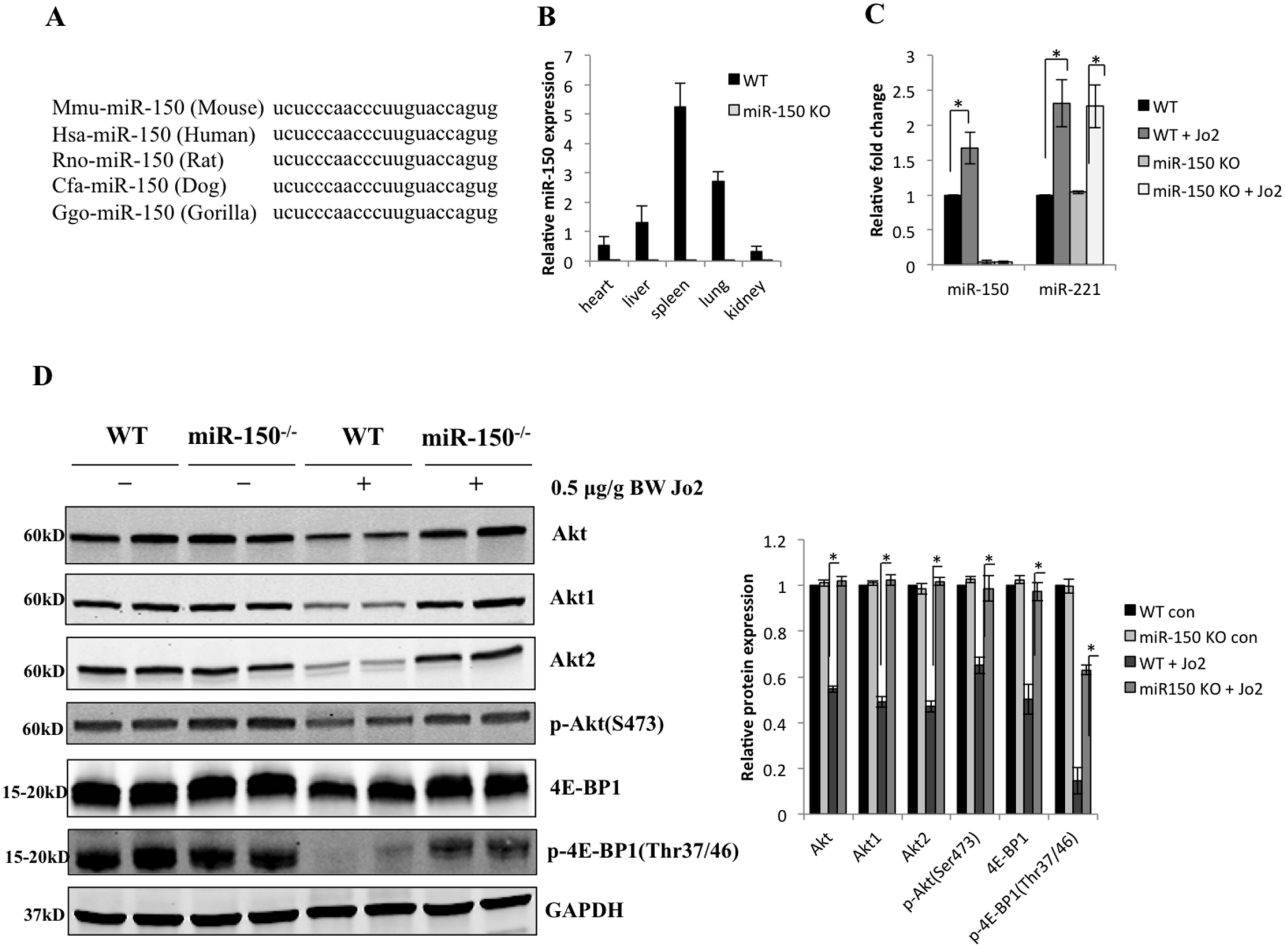
The levels of miR-150 and Akt in WT and miR-150 KO mice. (A) Mature miR-150 sequence was aligned among mouse, human, rat, dog and gorilla. (B) miR-150 expression in different tissues from WT and miR-150 KO mice. Total RNA was isolated from eight-week-old male WT and miR-150 KO mice. 1μg of total RNA was used for reverse transcription followed by qRT-PCR analysis. Relative expression of miR-150 was normalized to U6. Data are expressed as mean ± SD. (C) The levels of miR-150 and miR-221 in WT and miR-150 KO livers with or without Jo2 treatment. WT and miR-150 KO mice were intraperitoneally injected with or without 0.5 μg/g of body weight Jo2 (n = 6–8 per group). The livers were harvested 4 hours after Jo2 injection. The levels of miR-150 and miR-221 in the livers were determined by qRT-PCR (Data are expressed as mean ± SD, *p<0.05). (D) Western blot for Akt1, Akt 2, total Akt, p-Akt(Ser473), 4E-BP1 and p-4E-BP1(Thr37/46) (with GAPDH as the loading control). The blots shown in this figure were from two individual mice for each group. Quantifications of relative protein levels are shown at the right panel (the data are expressed as mean ± SD, *p<0.05).

Mature miR-150 contains 22 nucleotides; it is highly conserved through the species and has the same sequence among mice, human, rat, dog and gorilla (Fig 4A). We utilized qRT-PCR analysis to examine the expression of miR-150 in various tissues from the wild type and miR-150 KO mice. In the wild type mice, miR-150 is highly expressed in the spleen, moderately expressed in the lung, liver and heart, and slightly expressed in the kidney; as expected, miR-150 was undetectable in any of the tissues from the miR-150 KO mice (Fig 4B). We observed that Jo2 treatment induced approximately 1.7-fold increase of liver miR-150 in the WT mice; this phenomenon was not observed in the miR-150 KO mice (Fig 4C). Given that miR-221 is one of the highly upregulated miRNAs in response to Fas-induced apopotosis[31], we also examine the level of miR-221 in the livers; we observed a 2.3-fold increase in miR-221 expression in both WT and miR-150 KO mice after Jo2 injection (Fig 4C).

To identify the potential targets of miR-150, we utilized several bioinformatics prediction systems (including http://www.microRNA.org, http://www.targetscan.org, http://www.mirbase.org, and RNA22 microRNA prediction algorithm[26]). The search revealed that both *Akt1* and *Akt2* are predicted targets of miR-150 in mice, and Akt2 is predicted target of miR-150 in human. Noticeably, Akt is a family of serine/threonine protein kinase and important for protection against Fas-induced apoptosis[32–34]. Thus, we performed Western blotting analysis to determine the levels of Akt1, Akt2, total Akt and p-Akt(Ser437) in the livers of WT mice and miR150 KO mice with or without Jo2 treatment. As shown in Fig 4D, with Jo2 treatment the miR-150 KO livers expressed higher levels of Akt1, Akt2, total Akt and p-Akt(Ser473) than the WT livers, although under baseline condition (without Jo2 treatment), the miR-150 KO and WT livers express comparable levels of Akt1, Akt2 and total Akt. Given that eukaryotic translation initiation factor 4E-binding protein 1 (4E-BP1) is a downstream target of Akt (4E-BP1 negatively regulated eukaryotic translation initiation factor 4E [eIF4E], a key rate-limiting initiation factor for cap-dependent translation[35]), we further examined the level of 4E-BP1 and p-4E-BP(Thr37/46); we observed that both 4E-BP1 and p-4E-BP(Thr37/46) were higher in the miR-150 KO livers than the WT livers after Jo2 injection (Fig 4D). These observations suggest that upregulation of miR-150 during Fas induced liver injury may be a potential mechanism for decreased Akt activation in wild type mice.

Our bioinformatics analysis also reviewed other predicted targets of miR-150, including GSK-3β and cJun. However, we observed that the protein levels of GSK-3β and cJun did not differ between the WT and miR-150 KO livers (with or without Jo2 treatment) (S1 Fig).

### Inhibition of Akt restored Fas-induced liver injury in mice

**Fig 5:**
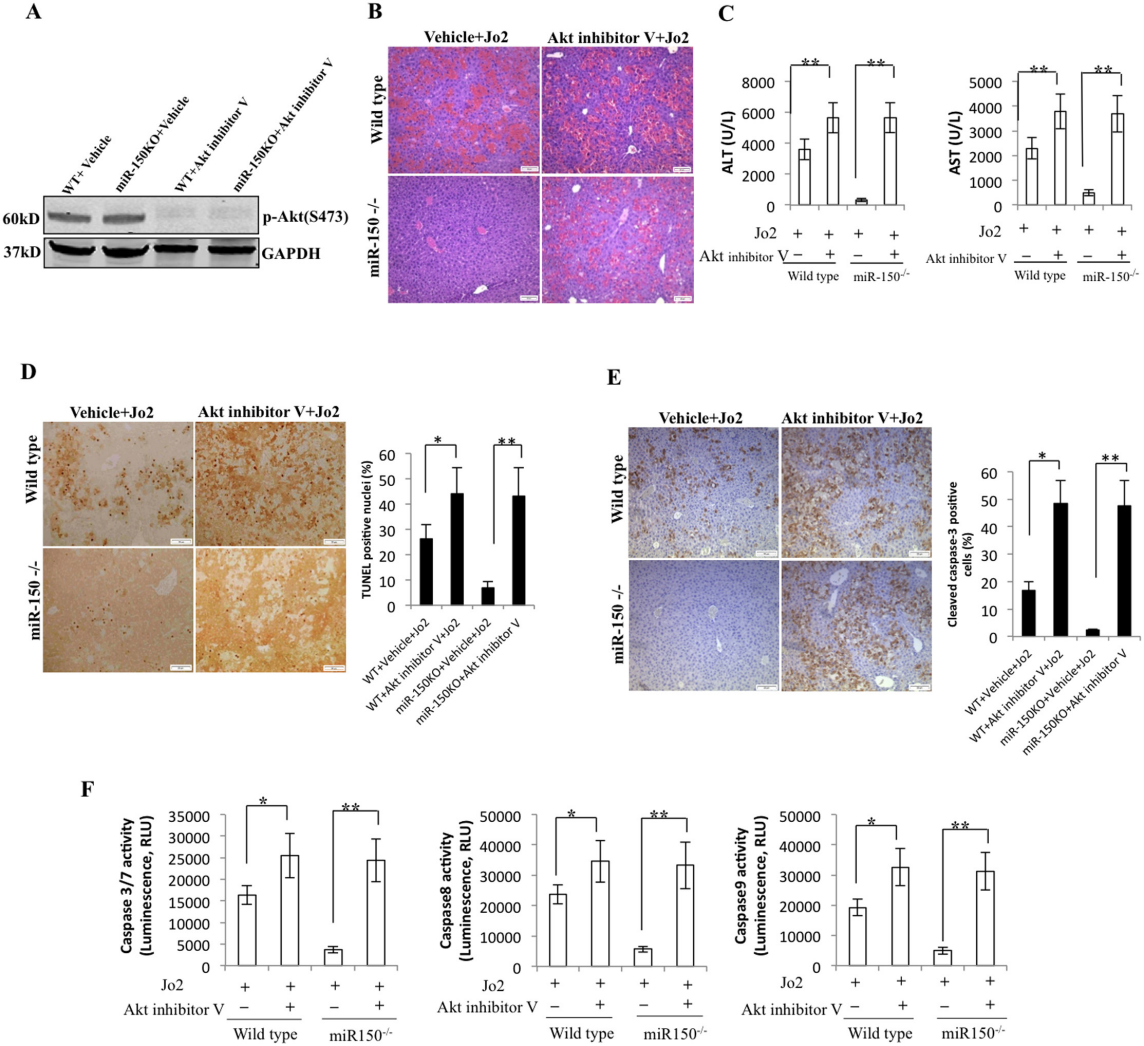
Effect of Akt inhibitor V on Fas-induced liver injury and hepatocyte apoptosis. (A) WT and miR-150 KO mice were injected intraperitoneally with vehicle or Akt inhibitor V (1mg/kg of body weight) 30 minutes before PBS administration (n = 3 each group). Mice were sacrificed 3 hours after PBS administration. Western blot showed that Akt inhibitor V treatment inhibited the phosphorylation of Akt. (B) WT and miR-150 KO mice were injected intraperitoneally with vehicle or Akt inhibitor V (1mg/kg of body weight) 30 minutes before Jo2 (0.5μg/g body weight) administration (n = 6 each group). Mice were sacrificed 3 hours after Jo2 administration. H&E staining (100×, scale bar 20μm) of formalin-fixed, paraffin-embedded liver tissues. (C) Serum levels of ALT and AST in mice with or without Akt inhibitor V pretreatment (data are expressed as mean ± SD **p<0.01). (D) TUNEL staining of the liver tissues from WT and miR-150 KO mice 3 hours after Jo2 injection (with or without Akt inhibitor V pretreatment). Representative photographs (100×, scale bar 20μm) are shown at the left panel; quantification of TUNEL-positive cells is shown at the right panel (the data are expressed as mean ± SD; *p<0.05, **p<0.01). (E) Immunostaining for cleaved caspase-3 in liver tissues from mice with or without Akt inhibitor V pretreatment (100×, scale bar 20μm). Quantification of cleaved caspase-3 positive cells is shown at the right panel (the data are expressed as mean ± SD; *p<0.05, **p<0.01). (F) Caspase-3/7, caspase-8, and caspase-9 activities in liver tissue from mice with or without Akt inhibitor V pretreatment (the data are expressed as mean ± SD; *p<0.05, **p<0.01).

To further investigate the role of Akt for protection against Fas-induced liver injury, we assessed the extent of Jo2-induced liver injury in WT and miR-150 KO mice pretreated with Akt inhibitor V (Triciribine). The efficacy of Akt inhibitor V was confirmed by the observation that Akt inhibitor V treatment inhibited the phosphorylation of Akt in mice without Jo2 treatment (Fig 5A). To evaluate the effect of Akt inhibitor V on Jo2-induced liver injury, WT and miR-150 KO mice were pretreated with vehicle or Akt inhibitor V (1 mg/kg) 30 minutes prior to the administration of Jo2 (0.5 μg/g body weight) or PBS, and the animals were sacrificed 3 hours after Jo2 injection. We observed that pretreatment with Akt inhibitor V reversed Jo2-induced liver injury in miR-150 KO mice, as documented by histopathological examination of the liver tissues (Fig 5B), analysis for serum transaminases (Fig 5C), TUNEL assay (Fig 5D), immunostain for cleaved caspase-3 (Fig 5E), as well as assessment for caspase-3/7, caspase-8 and caspase-9 activities (Fig 5F). These findings suggest an important role of Akt for protection against Fas-induced liver injury in miR-150 KO mice.

### The role of miR-150 in Fas-induced apoptosis of primary hepatocytes

**Fig 6:**
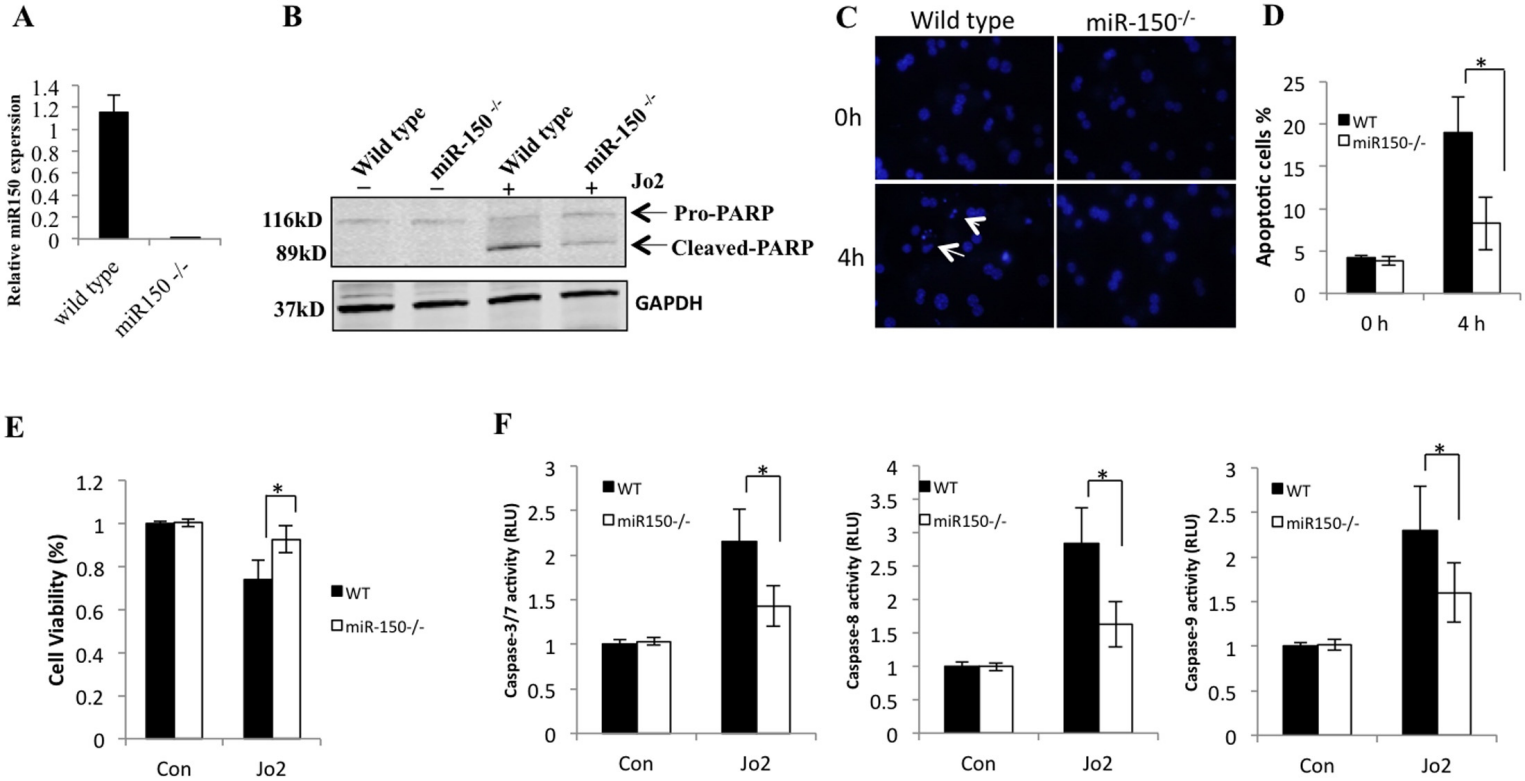
The role of miR-150 in Fas-induced primary hepatocyte apoptosis. Primary hepatocytes were isolated from eight-week-old male WT mice and miR-150 KO mice. (A) The expression of miR-150 in hepatocytes was quantified by qRT-PCR. Data are expressed as mean ± SD. (B) Hepatocytes were treated with Jo2 (0.5 μg/mL) plus CHX (10 μg/mL) for 4 hours. The cell lysates were obtained for Western blotting to detect PARP cleavage. (C) Representative Hoechst staining (200×) of WT and miR-150 KO hepatocytes 0 and 4 hours after treatment with 0.5 μg/mL Jo2 plus 10 μg/mL CHX. Arrows indicate fragmented nucleus. (D) Quantitative analysis for apoptotic cells under Hoechst staining. Data are expressed as mean ± SD, *p<0.05. (E) Cell viability as assessed by trypan blue exclusion 4 hours after treatment with 0.5 μg/mL Jo2 plus 10 μg/mL CHX or with an equal volume of 1×PBS plus DMSO as control (Con). The data are expressed as mean ± SD, *p<0.05. (F) Caspase-3/7, caspase-8, caspase-9 activities in WT and miR-150 KO hepatocytes 4 hours after treatment with 0.5 μg/mL Jo2 plus 10 μg/mL CHX or with an equal volume of 1×PBS plus DMSO as control (Con). The results are expressed as mean ± SD of fold changes over WT hepatocytes.

Hepatocytes are sensitive to Fas-mediated apoptosis due to their high constitutive expression of Fas[36]. To determine the contribution of hepatocyte miR-150 in Fas-mediated apoptosis, primary hepatocytes were isolated from WT mice and miR-150 KO mice; the cultured hepatocytes were treated with Jo2 plus CHX to determine parameters of apoptosis. As expected, qRT-PCR analysis showed that miR-150 is expressed in the hepatocytes isolated from the wild type mice, but not in hepatocytes isolated from miR-150 KO mice (Fig 6A). Jo2 treatment induced less prominent PARP cleavage in miR-150 KO hepatocytes compared to wild type hepatocytes (Fig 6B). Likewise, Jo2 treatment induced less prominent nuclear fragmentation in miR-150 KO hepatocytes compared to wild type hepatocytes (analyzed by Hoechst staining, Fig 6C and 6D). The viability of hepatocytes was higher in miR-150 KO hepatocytes than in the WT hepatocytes after Jo2 treatment (Fig 6E). Furthermore, Jo2-induced increase of caspase-3/7, caspase-8, caspase-9 activities was less prominent in miR-150 KO hepatocytes compared to wild type hepatocytes (Fig 6F). These findings provide direct evidence for miR-150 in hepatocytes for protection against Fas-induced apoptosis.

### miR-150 directly targets *Akt1* and *Akt2* in hepatocytes

**Fig 7:**
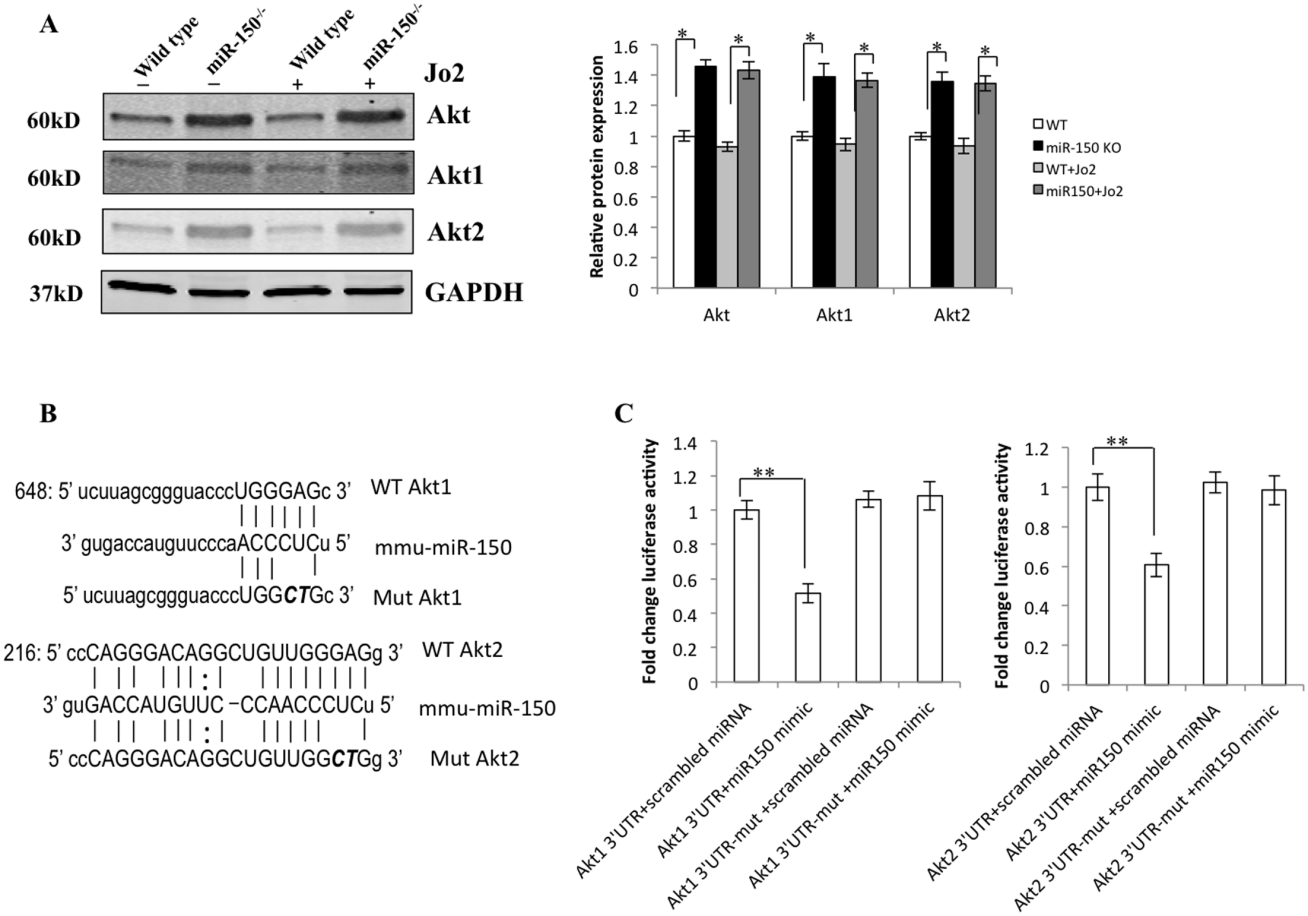
miR-150 targets *Akt1* and *Akt2* in hepatocyte. (A) Primary hepatocytes isolated from WT mice and miR-150 KO mice were treated with Jo2 (0.5 μg/mL) for 4 hours and the cell lysates were subjected to Western blotting for Akt1, Akt2 and total Akt. Quantifications of relative Akt1, Akt 2 and total Akt protein levels are shown at the right panel. Data are expressed as mean ± SD *p<0.05. (B) The 3’-UTRs of *Akt1* and *Akt2* contain predicted miR-150 binding sites. Mutations were generated on the potential targets sequences as indicated. (C) Luciferase reporter assay. Hepatocytes were isolated from WT mice and cotransfected with miR-150 mimic plus the reporter plasmid containing the 3’-UTR of *Akt1*, *Akt2* or their mutants. The experiments were repeated three times. Data are expressed as mean ± SD, **p<0.01.

The basal levels of Akt1, Akt2 and total Akt were higher in the miR-150 KO hepatocytes compared to the WT hepatocytes (Fig 7A). These findings suggest that the levels of Akt1 and Akt2 in hepatocytes are influenced by miR-150. As *Akt1* and *Akt2* each contain a single miR-150 binding site in 3’-UTR, we generated reporter constructs containing 3’-UTR of *Akt1* and *Akt2* with mutation of the miR-150 binding site (indicated in Fig 7B). The *Akt1* and *Akt2* 3’-UTR or their corresponding mutants were transfected into the primary hepatocytes isolated from the wild type mice, with cotransfection of miR-150 mimic, to determine luciferase reporter activity. As shown in Fig 7C, miR-150 mimic significantly decreased the 3’-UTR luciferase reporter activity of *Akt1* and *Akt2*; this effect was abolished when the miR-150 binding site was mutated. These observations demonstrate that both *Akt1* and *Akt2* are direct targets of miR-150 in mouse primary hepatocytes.

### Delivery of miR-150 enhances Fas-induced apoptosis in miR-150 KO mice

**Fig 8:**
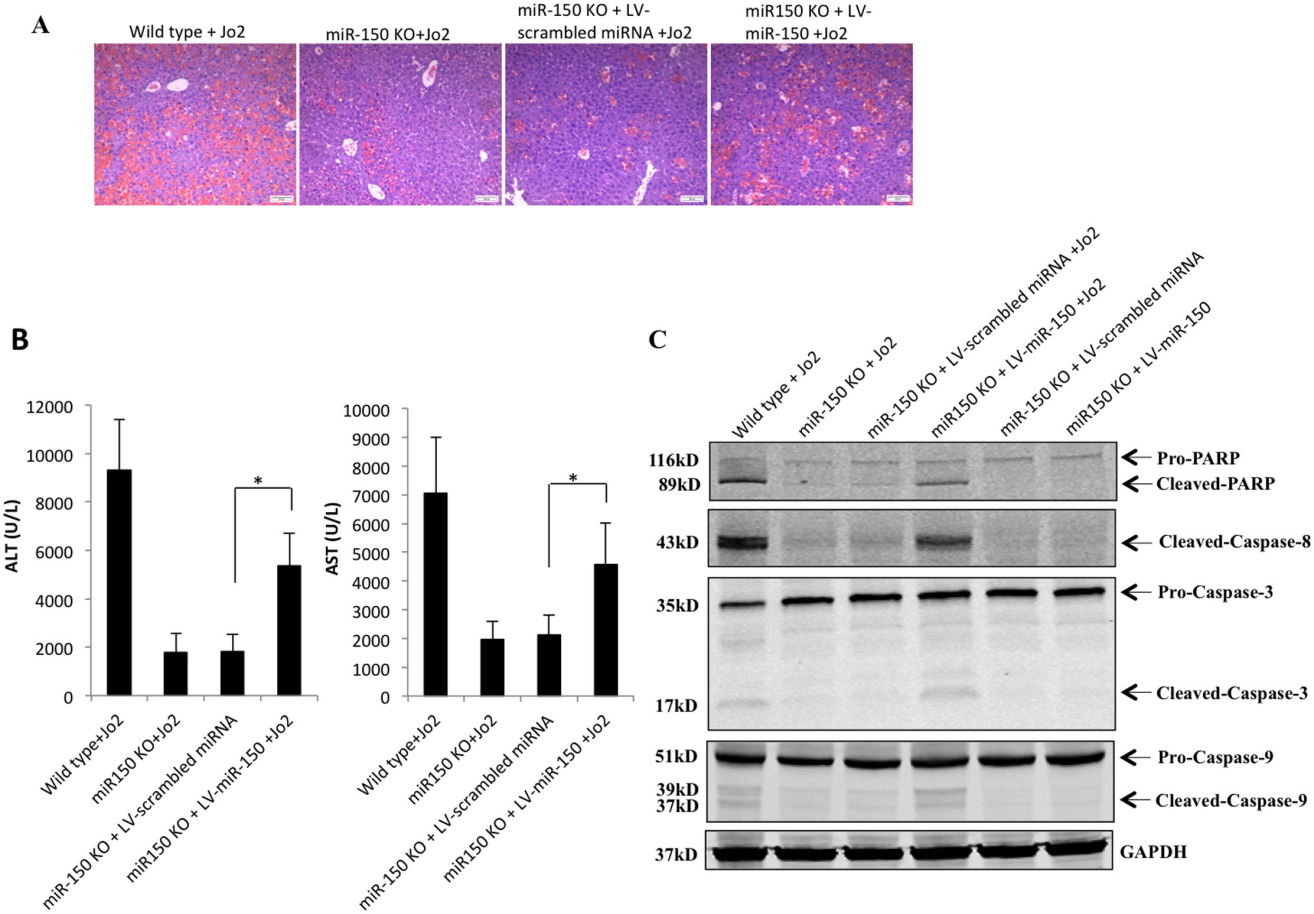
Restoration of miR-150 expression enhances Fas-induced apoptosis in miR-150 KO mice. Eight-week-old miR-150 KO mice were administrated lentiviral particles containing pre-*miR-150* (LV-*miR-150*, 1.01×10^9^ copies/mouse) (n = 6) or lentiviral particles containing scrambled miRNA (LV- scrambled-miRNA, 4.8×10^8^ copies/mouse) (n = 6) by tail vein injection in a volume of 200 μL of sterile saline. 7 days after lentiviral particles injection, the mice were treated with 0.5 μg/g of body weight Jo2 for 4 hours. (A) Hematoxylin and eosin (H&E) staining of the liver tissues (100×, scale bar 20μm). (B) Serum ALT and AST levels. Data are expressed as mean ± SD, *p<0.05. (C) Western blot analysis for the cleavage of PARP and caspases.

To further evaluate the role of miR-150 in Fas-induced liver injury, we utilized lentiviral vector to restore miR-150 expression in miR-150 KO mice. Specifically, 8 week-old miR-150 KO mice were administered lentiviral particles containing pre-*miR-150* (LV-*miR-150*) or scrambled control miRNA (LV-scrambled-miRNA) by tail vein injection. In this system, delivery of pre-*miR-150* or control lentiviral particles did not alter liver tissue histology or transaminase levels (S2 Fig). Successful hepatic expression of miR-150 in mice injected with pre-*miR-150* lentiviral particles was confirmed by qRT-PCR analysis (approximately 60 fold increase, see S2 Fig). Fluorescence microscopy revealed transduction of hepatocytes by the lentiviral particles (S2 Fig). 7 days after virus injection, the mice were intraperitoneally administered Jo2 (0.5 μg/g of body weight). We observed that LV-*miR-150* injection enhanced Jo2-induced liver injury, as reflected by more evident tissues damage and more prominent increase in serum transaminases, caspase activation and PARP cleavage in comparison to injection of LV-scrambled control miRNA (Fig 8). Thus, delivery of miR-150 to miR-150 KO mice is able to restore Fas-induced liver injury, *in vivo*.

## Discussion

Fas is a cell surface protein that belongs to the tumor necrosis factor receptors superfamily[37]. Stimulation of Fas by its ligand causes rapid assembly of the death-inducing signaling complex (DISC), which consists of Fas receptor, FADD (Fas-associated death domain protein) and caspase-8[36, 38, 39]. Fas triggers apoptosis through FADD-mediated recruitment and activation of caspase-8 to the DISC where they are autocatalytically activated then starting a caspase-dependent signaling cascade. In this study, we demonstrate that miR-150 deficiency protects against Fas-induced hepatocyte apoptosis *in vivo* and *in vitro*. The miR-150 KO mice showed lower mortality, lower aminotransferase levels, less liver tissue damage, fewer apoptotic hepatocytes and lower caspase activities after Jo2 treatment. The role of miR-150 in Jo2-induced liver injury was further supported by the fact that lentiviral delivery of miR-150 enhanced Jo2-induced liver injury in miR-150 KO mice. The primary hepatocytes isolated from miR-150 KO mice also showed resistance to Fas-induced apoptosis compared to wild type hepatocytes. Our findings provide novel evidence for an important role of Akt in protection against Fas-induced hepatocyte apoptosis in miR-150 KO mice.

Akt (also known as protein kinase B [PKB]) is one of the key molecules downstream of the phosphoinositide 3-kinase (PI3K) signaling pathway. In mammals, Akt comprises of three highly homologous members, including Akt1 (PKBα), Akt2 (PKBβ) and Akt3 (PKBϒ), which are encoded by three different genes located on different chromosomes[40–43]. The Akt kinases control an array of diverse functions including cell growth, survival, proliferation and metabolism[44]. Akt1 and Akt2 are widely expressed, whereas Akt3 expression is restricted to brain, testis, lung, fat, mammary glands and pancreatic islets[43]. In the liver, only Akt1 and Akt2 but not Akt3 is expressed, with Akt2 as the major isoform (accounting for approximately 70% of total Akt protein)[43]. Several growth factors and cytokines are known to confer resistance to Fas-induced liver injury by activation of the Akt pathway[45, 46]. In our study, the role of Akt in miR150 deficiency-mediated resistance to Fas-induced hepatocyte apoptosis is supported by the following observations: (1) the protein levels of Akt1 and Akt2 are higher in miR-150 KO hepatocytes and liver tissues; (2) miR-150 directly targets the 3’UTR of *Akt1* and *Akt2* in hepatocytes; (3) inhibition of Akt restores Fas-induced hepatocyte apoptosis and liver injury in miR-150 KO mice.

We did not observe difference in Akt1 and Akt2 levels in WT and miR-150 KO livers under basal conditions (without Jo2 treatment). However, we observed different Akt1 and Akt2 levels in miR-150 KO hepatocytes without Jo2 treatment. The exact reason for different Akt expression between liver tissue homogenates and cultured primary hepatocytes under basal conditions (without Jo2 treatment) is not known and remains speculative, although one possibility may relate to the expression of Akt in other cell types. While the current study details the regulation of Akt1/2 by miR-150 in hepatocytes, it remains unknown whether miR-150 may also regulate the expression of Akt in other cell types in the liver. Given that it is well known that one gene can be regulated by multiple miRNAs, it remains to be determined whether the expression of Akt in the liver may also be influenced by other miRNAs.

Our findings suggest that miR-150 modulates liver injury depending on the context of the liver injuries. Although miR-150 deficiency protects the liver from Fas-induced apoptosis, it did not prevent LPS/D-GalN-induced liver injury. Previous studies have shown that the plasma levels of miR-150 are decreased in patients with sepsis[22] and that low serum miR-150 levels are associated with an unfavorable prognosis of septic patients[23]. In the LPS/D-GalN model, D-GalN blocks gene transcription in the liver and LPS in turn induces an acute cytokine-dependent liver inflammation accompanied by massive liver apoptosis and death of the animals[24, 47, 48]; our results in the current study suggest that miR-150 is not essential in these processes. On the other hand, our data support a key role of miR-150 in Fas-induced hepatocyte apoptosis; this observation is noteworthy, given that Fas-induced apoptosis is implicated in the pathogenesis of hepatitis and hepatic failure[31, 49–51]. For example, viral hepatitis from hepatitis C virus (HCV) and hepatitis virus (HBV) are known to increase Fas expression whose levels correlate with disease activity and response to therapy[47]. In chronic hepatitis C patients, Fas and Fas ligand are elevated and Fas-induced apoptosis is one of the mechanisms for HCV-induced hepatocytes apoptosis[52, 53]. HBV is known to sensitize hepatocytes to Fas signaling and this alteration may contribute to hepatocarcinogenesis[54]. Fas signaling pathway also plays important roles in alcoholic liver disease[55], non-alcoholic steatohepatitis[56] and cholestatic liver injury[57]. Thus, modulation of Fas-induced hepatocyte apoptosis by miR-150 may have broad implication in various acute and chronic liver diseases.

It has been recognized that human liver regeneration may be orchestrated by distinct miRNAs, which may control key processes of liver regeneration including hepatocyte proliferation[58]. Noticeably, inhibition of several microRNAs including miR-150 is associated with enhanced cell proliferation and successful liver regeneration[58]. The latter observation is corroborated by the finding in our current study that miR-150 deficiency protected against Fas-induced hepatocyte apoptosis and liver injury through activation of Akt. Thus, inhibition of miR-150 may confer pro-proliferative and anti-apoptotic effects which may facilitate the repair of liver injuries.

In summary, this study provides the first evidence that miR-150 deficiency protects against Fas-induced hepatocyte apoptosis and liver injury through upregulation of the Akt pathway. Our findings point toward a key role of miR-150 in hepatocytes for regulation of the Fas apoptotic pathway and suggest the possibility of inhibiting miR-150 for liver injury repair. Given the emerging function of miR-150 in liver parenchymal and nonparenchymal cells, further studies are warranted to detail its mechanism of actions in liver cells and liver diseases.

## Supporting Information

S1 Fig The levels of GSK-3β and c-Jun in WT and miR-150 KO livers. (TIF)

S2 Fig Lentiviral delivery of miR-150. (TIF)
