# Supplementary figures and images for: miR-150 Deficiency Protects against FAS-Induced Acute Liver Injury in Mice through Regulation of AKT
Source: PLoS One. 2015 Jul 21;10(7):e0132734. doi: 10.1371/journal.pone.0132734 (PMC4510058; doi:10.1371/journal.pone.0132734)

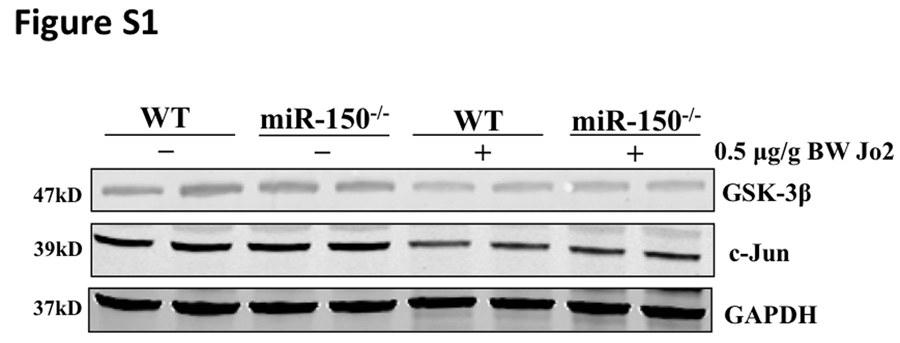

Supplement: S1 Fig — (TIF) [file pone.0132734.s001.tif]

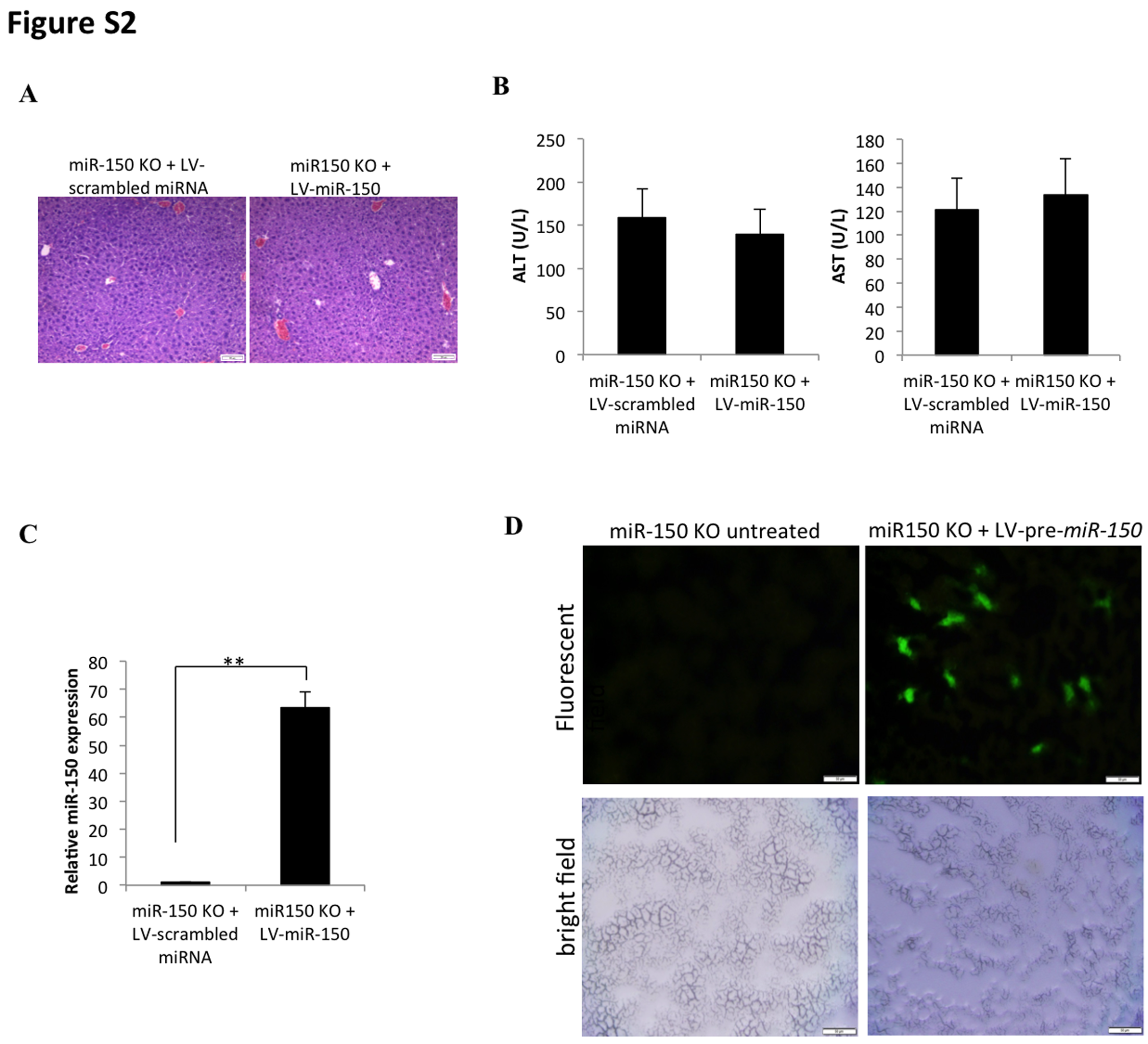

Supplement: S2 Fig — (TIF) [file pone.0132734.s002.tif]
